# Uptake of inorganic mercury by human locus ceruleus and corticomotor neurons: implications for amyotrophic lateral sclerosis

**DOI:** 10.1186/2051-5960-1-13

**Published:** 2013-05-09

**Authors:** Roger Pamphlett, Stephen Kum Jew

**Affiliations:** 1 The Stacey Motor Neuron Disease Laboratory, Department of Pathology, Sydney Medical School, The University of Sydney, New South Wales, Australia

**Keywords:** Amyotrophic lateral sclerosis, Motor neuron disease, Mercury, Neurotoxin, Corticomotor neuron, Locus ceruleus, Stress, Exercise

## Abstract

**Background:**

Environmental toxins are suspected to play a role in the pathogenesis of amyotrophic lateral sclerosis (ALS). In an attempt to determine which pathways these toxins can use to enter motor neurons we compared the distribution of mercury in the CNS of a human and of mice that had been exposed to inorganic mercury.

**Results:**

In the human who had been exposed to metallic mercury, mercury was seen predominantly in the locus ceruleus and corticomotor neurons, as well as in scattered glial cells. In mice that had been exposed to mercury vapor or mercuric chloride, mercury was present in lower motor neurons in the spinal cord and brain stem.

**Conclusions:**

In humans, inorganic mercury can be taken up predominantly by corticomotor neurons, possibly when the locus ceruleus is upregulated by stress. This toxin uptake into corticomotor neurons is in accord with the hypothesis that ALS originates in these upper motor neurons. In mice, inorganic mercury is taken up predominantly by lower motor neurons. The routes toxins use to enter motor neurons depends on the nature of the toxin, the duration of exposure, and possibly the amount of stress (for upper motor neuron uptake) and exercise (for lower motor neuron uptake) at the time of toxin exposure.

## Background

The cause of sporadic amyotrophic lateral sclerosis (SALS) remains unknown [1]. Debate continues as to whether SALS is due to rare gene mutations (either germline or somatic), or is due to gene-environment interactions that involve toxins or viruses. Of note in this regard, in a recent study 11% of SALS patients had mutations in one of 6 known ALS genes, compared to 67% of familial ALS (FALS) patients [2]. If this FALS-SALS percentage ratio were to remain the same when 100% of FALS-causing mutations are discovered, by extrapolation only 16% of SALS patients will harbour ALS-causing mutations. That leaves 84% of SALS patients whose disease needs to be explained by some other mechanism. The percentage of patients with true SALS may be even more than previously believed, since the occurrence of ALS in distant relatives (who are then considered to be members of FALS kindreds) may be by chance [3]. One meta-analysis arrived at a figure for FALS of 5% of total ALS patients, while another (which divided FALS into definite, probable and possible categories) indicated that FALS due to penetrant mutations is likely to represent no more than 10% of total ALS patients [4].

An environmental toxin that enters motor neurons selectively, and then either alone, or in combination with some genetic susceptibility to the toxin, destroys the motor neuron could underlie SALS [5]. Heavy metals in particular have been suspected to be involved in ALS [6]. However, despite a plethora of epidemiological, experimental and pathological studies, evidence to back this hypothesis has been hard to come by. Some support comes from studies showing that neurotoxins given systemically to animals localise preferentially to motor neurons [7], that certain occupations appear to be associated with an increased risk of SALS [8], and that gene-environment interactions involving neurotoxins may underlie some cases of SALS [9]. A difficulty in looking for SALS-causing toxins in human tissue is that by the time of death most motor neurons in SALS have disappeared and thus are not available for examination. Furthermore, the few remaining SALS motor neurons may still be present because they did not take up the toxin in the first place, a so-called a “survivor” effect. Conversely, damaged motor neurons may take up metals secondarily to the disease process and so confuse the issue [10]. At present only a few toxins (all of them heavy metals) can be identified histologically [11]. Finally, it is rare to obtain CNS tissue for histological examination from humans who have been exposed to known toxins. For example, there are only three previous reports of humans exposed to inorganic mercury whose CNS tissue has been studied with modern histological techniques [12-14].

In 1996 we reported the distribution of mercury in the CNS of a man who injected himself with metallic mercury and who then committed suicide shortly afterwards [14]. At *post mortem*, mercury deposits were found in a number of sites in the CNS, particularly in corticomotor neurons (CMNs). However, no explanation for this CMN uptake of mercury was offered. The mercury-detection technique used in this study was autometallography (AMG) [15], and sections were kept in the staining reagents for varying lengths of time to demonstrate the mercury grains better in cells where mercury concentrations were low. This meant, however, that comparisons between the amount of mercury in different neurons could not be made. We have now undertaken three further investigations using tissue from this mercury-exposed human, as well as tissue from mercury-exposed mice, in an attempt to gain further insights into the relationship between toxins and ALS.

(1) We have re-stained tissue from the mercury-exposed human, using a standardised AMG protocol that enables us to determine which neurons take up the mercury most avidly. From the distribution of these mercury-containing neurons we suggest a pathway via the locus ceruleus (LC) that circulating mercury may use to enter CMNs, and propose that this uptake may be upregulated by stress.

(2) There has been much interest in the role of glia in ALS [16], as well as in the role astrocytes play in mercury CNS toxicity [17]. Although glial uptake of mercury was noted in passing in our previous report [14], the distribution of mercury-containing glia in the brain was not studied. We have therefore mapped the regions of the brain in which glial uptake of mercury was present in an attempt to assess the role of mercury-containing glia in motor neuron damage.

(3) Differences between the uptake of inorganic mercury in humans and rodents has previously been noted, with rodents having prominent lower motor neuron (LMN), but negligible CMN and glial uptake of this toxin. No direct comparisons between human and rodent uptake of inorganic mercury have however been undertaken. We have therefore re-stained tissue from mercury-exposed mice and compared the distribution of mercury in these mice with mercury-exposed human tissue. This has enabled us to outline different pathways that neurotoxins such as mercury may use to enter motor neurons. We propose that these different routes of toxin uptake into CMNs and LMNs could explain the phenotypic variation that is such a prominent feature of ALS.

## Results

### Distribution of mercury in the human CNS after exposure to metallic mercury

**Figure 1:**
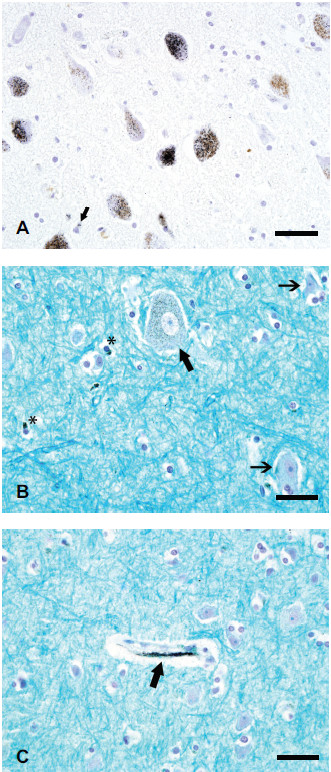
**Mercury staining in human brain after exposure to metallic mercury.** (**A**) Mercury in locus ceruleus neurons is seen as small black Hg^AMG^ grains, which can readily be distinguished from the pale yellow-brown neuromelanin granules in the cytoplasm. An occasional glial cell (arrow) contains a few Hg^AMG^ grains. (**B**) The cytoplasm of this corticomotor neuron (thick arrow) in the frontal motor cortex contains numerous Hg^AMG^ grains. Two nearby small neurons (thin arrows) contain no mercury. The cytoplasm of some glia (asterisks) contains mercury. (**C**) Mercury staining is seen in the region of this capillary wall (arrow) in the frontal motor cortex. No adjacent small neurons or glia contain mercury. LFB-Hg^AMG^, bar = 50 μm.

1. *Heavy and widespread mercury staining*. This was present in only two groups of neurons: (a). Locus ceruleus (LC) neurons contained the heaviest concentration of mercury in the brain. About 70% of these neurons contained autometallography-demonstrable mercury (Hg^AMG^) grains, which were widespread within the cytoplasm of affected cells (Figure 1A). The small black Hg^AMG^ grains could readily be distinguished from the larger pale brown granules of neuromelanin. The total number of neurons within the LC appeared normal, though no formal quantitation was undertaken. (b). CMNs in the frontal motor strip stood out clearly on AMG staining because of the heavy concentration of mercury in all visible CMNs (Figure 1B). No adjacent smaller neurons, or large neurons in the somatosensory cortex, contained Hg^AMG^ grains.

2. *Heavy and patchy mercury staining*. Hg^AMG^ grains were seen in subependymal astrocytes adjacent to all ventricles.

3. *Light and widespread mercury staining*. Capillaries in all parts of the brain contained patchy mercury staining, either in the wall itself or adjacent to the wall (Figure 1C).

4. *Light and patchy mercury staining*. Light-patchy Hg^AMG^ grains were seen in: (a) cerebellar dentate nucleus neurons; (b) scattered individual neurons in the brain stem reticular formation and periaqueductal grey matter; and (c) neurons in the hypothalamus, vestibular nucleus, and nucleus intercalatus. Pineal and choroid plexus cells also contained light-patchy Hg^AMG^ grains.

5. *Glial cells*. Scattered glial cells contained Hg^AMG^ grains. These were not distributed evenly throughout the brain, but were concentrated in the frontal motor cortex (Figure 1B), red nucleus, pallidum, caudoputamen, and cerebellar dentate nucleus. The round, pale, nuclear morphology of the cells suggested that most of these cells were astrocytes. The mercury was situated adjacent to the nucleus and not obviously in any processes.

**Figure 2:**
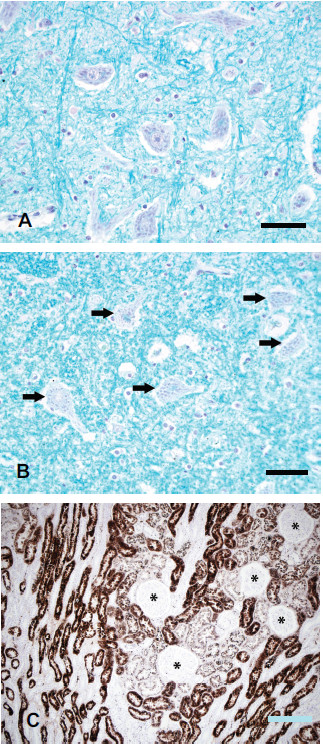
**Results of mercury staining in human lower motor neurons and kidney after exposure to metallic mercury.** (**A**) No stainable mercury is present in hypoglossal motor neurons or in nearby glia. LFB-Hg^AMG^, bar = 50 μm. (**B**) No stainable mercury is present in C3 anterior horn spinal motor neurons (arrows) or surrounding glia. LFB-Hg^AMG^, bar = 50 μm. (**C**) Heavy mercury staining is present in renal tubules, but not in glomeruli (asterisks). Hematoxylin-Hg^AMG^, bar = 200 μm.

No mercury staining was seen in neurons in motor nuclei of the brain stem, including the hypoglossal nucleus (Figure 2A), in motor neurons in spinal lamina 9 down to the C3 level (Figure 2B), or in the substantia nigra (including the neuromelanin-containing neurons).

In a non-mercury-exposed control 26 year-old male individual no mercury staining was seen in neurons or glia from any of the above regions.

### Distribution of mercury in mouse CNS

**Figure 3:**
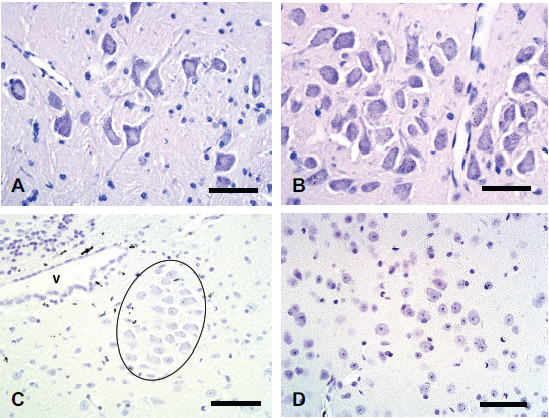
**Mercury staining in mouse spinal cord and brain after exposure to mercury.** (**A**) After Hg^0^ exposure, all large spinal motor neurons in the cervical spinal cord contain Hg^AMG^ grains. No glial staining is seen. (**B**) After Hg^0^ exposure, all large hypoglossal motor neurons contain Hg^AMG^ grains. No glial staining is seen. (**C**) After Hg^0^ exposure, dense Hg^AMG^ grains are seen in subependymal astrocytes near the ventricle (V), but no locus ceruleus neurons (outlined) contain mercury. (**D**) After mercuric chloride exposure, no mercury staining is present in layer 5 somatomotor neurons in the frontal lobe, or in surrounding glial cells. Hematoxylin-Hg^AMG^, bar = 50 μm.

The distribution of mercury in the CNS of mice that had been exposed to either mercury vapor (Hg^0^) or mercuric chloride (HgCl_2_) was similar to that previously described in rodents [18-20]. In all mice, heavy widespread mercury staining was seen in lamina 9 motor neurons in the spinal grey matter (Figure 3A). All brain stem cranial nerve motor neurons stained heavily with mercury, particularly the hypoglossal nucleus (Figure 3B) and nucleus ambiguus, but also the extraocular muscle motor nuclei. Slight patchy mercury staining was present in the dorsal motor nucleus of the vagus nerve. Subependymal cells stained prominently for mercury (Figure 3C). Scattered capillary walls contained Hg^AMG^ grains in mice sacrificed 7 days after mercury exposure (but not after longer times after exposure), as did a few ependymal cells. No mercury staining was seen in mouse LC neurons (Figure 3C) or in neurons in somatomotor layers 3 or 5 (Figure 3D). No mercury staining was seen in glial cells of the brain or spinal cord in any mice.

### Kidney mercury staining

1. *Human*. Heavy mercury staining was present in renal tubules, but not in glomeruli, of the mercury-exposed human (Figure 2C).

2. *Mouse*. Mercury staining was present in renal tubules of mice sacrificed 7 days after exposure to either HgCl_2_ or Hg^0^, but not in mice sacrificed 705 days after HgCl_2_ exposure. To see if kidney mercury might be transferred via the sympathetic nervous system to CMNs in the same way as rabies virus [21], sections from the intermediolateral neurons in the thoracic spinal cord and from the thoracic sympathetic chain were stained with AMG. No intermediolateral neurons, and only about 1% of sympathetic chain neurons, contained light Hg^AMG^ staining.

## Discussion

Mercury was deposited predominantly in CMN and LC neurons of an individual who had injected himself with metallic mercury. The heavy deposition of mercury in human LC neurons suggests that these cells play a part in the selective uptake of inorganic mercury by CMNs, since these cells synapse with each other. This human distribution of mercury differed from that seen in mice exposed to inorganic mercury, where mercury was found in brain stem and spinal motor neurons. This suggests either that uptake of the toxin differs in humans and rodents, or that differences in the chemical forms of this toxin, duration of exposure to the toxin, or other factors that increase uptake mechanisms of the toxin, result in the toxin being taken up by different groups of motor neurons.

### Uptake of metals by corticomotor neurons

#### Human

AMG has been used to study the distribution of inorganic mercury in the brain of two other humans. The first was a man aged 50 years at death who had worked for 18 months filling mercury thermometers before presenting 16 years later with tremor and dysarthria [12]. After his death mercury staining was seen in some large neurons in the cerebral cortex, but whether or not these were CMNs cannot be determined. The second individual was a man who had been exposed to Hg^0^ for 13 years when he recycled mercury from amalgams, with an acute exposure to Hg^0^ when aged 41 years [13]. He had no specific symptoms of mercury toxicity, and died in 1990 from lung cancer, not having been at work for 16 years. He had widespread neuronal mercury staining, but no mention of mercury in CMNs was made. Therefore our case is the only one in which human CMN mercury can confidently be said to be present. In our dissection protocol, the cerebral hemispheres are first cut in the horizontal plane from the vertex for 40 mm, before the remainder of the hemispheres are sectioned coronally [22]. This allows us to obtain long strips of primary motor cortex that contain large numbers of CMNs, which makes the assessment of mercury distribution within these neurons straightforward.

#### Animal

None of our mercury-exposed mice had stainable mercury within their CMNs. CMN uptake of metals in other animal studies has varied. After mice were exposed to a large dose of bismuth subnitrate, large cell bodies in the frontal cortex (probably CMNs) stained positively with AMG [23]. In rats given intraperitoneal HgCl_2_ a few cells in cortical lamina 6 stained with AMG [18], but no cortical staining was seen in rats given oral HgCl_2_[24]. Mercury-sensitive SJL/N mice exposed long-term to Hg^0^ had stainable mercury in neocortical layer 5 [25], unlike short-term Hg^0^ exposure of non-mercury sensitive Wistar rats where staining was in layer 3 [19]; this suggests that genetic background and length of mercury exposure may influence which neocortical cells take up inorganic mercury. In squirrel monkeys exposed to a large dose of Hg^0^, the giant pyramidal cells of the precentral cortex contained more mercury than other neurons, though neuronal uptake was widespread at these doses [26].

Differences between mouse and human CMNs could underlie the lack of mercury uptake in mouse CMNs. Mice do not have a subpopulation of very large CMNs that are the equivalent of human Betz cells, and mouse CMNs do not make direct synaptic contact with LMNs as is the case with humans [27]. Mice may not be subjected to the long-term stressors that humans experience, and so may not be prone to take up toxins via the locus ceruleus pathway (see later). The resistance of rodent CMNs to toxin uptake could be one reason that rodent models of ALS have a relatively poor track record when it comes to translating therapeutic trials from rodents to humans [28].

### Uptake of metals by locus ceruleus neurons

#### Human

The two previous AMG studies on mercury-exposed humans do not mention whether or not the LC contained mercury [12,13]. It is therefore only our case in which mercury uptake in the LC has been demonstrated. One reason for this may be that our individual would have been under considerable stress, and this may have prompted mercury uptake into CMNs via the LC (see below).

#### Animal

We did not see any LC uptake of mercury in our mercury-exposed mice. The only report of uptake of a metal by the LC in rodents was a scanty uptake after mice were given a large toxicity-inducing dose of intraperitoneal bismuth subnitrate [23]. After this large dose of bismuth, many large neurons, including those in layer 5 of the frontal and parietal cortex, contained this metal [23]. LC neuronal uptake was not mentioned in rats given intraperitoneal HgCl_2_[18], rats given oral HgCl_2_[24], rats exposed to Hg^0^[19], or mice exposed to Hg^0^[25]. In most of these reports the LC was not mentioned, which may be because it contained none of the metal or because this is very small nucleus in rodents and can easily be overlooked. Rats exposed to a very high dose of Hg^0^ did have mercury in their LC, but with this high circulating level of mercury neuronal uptake was widespread [29]. In a report of mercury-exposed primate CNS that was examined with AMG the LC was not mentioned [30].

### Uptake of metals by glia

#### Human

In one previous mercury-exposed human, Hg^AMG^ staining was seen in astrocytes, but the distribution of these astrocytes was not given [12]. In our case, astrocytes in various sites contained mercury. There did not appear to be any particular pattern to this distribution, and the relationship between mercury-containing glia and neurons was weak. The only region where mercury-containing glia were consistently found adjacent to mercury-containing neurons was in the frontal motor cortex: here all mercury-containing CMNs had nearby astrocytes that also contained mercury. Astrocytes appear to have different domains within the CNS [31] so some groups of astrocytes may have more of a propensity to take up metals than others. However, it seems more likely that these mercury-astrocytes are attempting to lower the mercury burden of the CMNs by removing some of the metal from these neurons [12,17]. If this is the case, the CMN lays itself open to being subject to a perverse toxic-gain-of-function if the mercury in the astrocyte then causes an excitotoxic insult to the CMN via excess glutamate [32-34] or via the onset of neuroinflammation [35]. Finally, it is possible mercury is moving in the opposite direction, with astrocytes transferring mercury they have taken up from blood vessels to CMNs. The exact role of glial heavy metals in human neurotoxicology is thus not yet clear.

#### Animal

Uptake of metals by rodent astrocytes and microglia has been noted to be much less prominent than uptake into primate and human glia [17]. We too found this to be the case in our mice exposed to HgCl_2_ or Hg^0^ since no glia in the CNS contained significant amounts of mercury. This may explain why rodents appear to be relatively resistant to the major neurotoxic effects of inorganic mercury [36], since there would be no mercury in astrocytes to provoke a glutamate-induced excitotoxic attack on nearby neurons [34].

### Routes of metal transport into corticomotor neurons

#### Via the locus ceruleus

The LC has a potential exposure to circulating toxins through its extensive innervation of CNS blood vessels, and has extensive collateral axonal innervation to many CNS neurons, including CMNs [37]. Although a route for mercury from intracerebral blood vessels via the LC to CMNs has not yet been proven experimentally, a potential pathway would be: (1) metallic mercury in tissues releases Hg^0^ which is converted into Hg^2+^[38]; (2) Hg^2+^ enters the circulation and is bound to thiol-containing molecules such as cysteine [39]; (3) the Hg^2+^-thiol complexes in the region of capillaries are taken up at LC axon terminals, probably by some form of molecular mimicry. This could be by using the re-uptake of noradrenaline (NA) into LC terminals through the norepinephrine transporter, since NA axon terminals are intimately associated with intraparenchymal blood vessels [40]; (4) the Hg^2+^-thiol complex enters the axon of the LC and is transported via retrograde axonal transport towards the cell body; (5) some Hg^2+^ remains in the LC cell body, but some is transferred via anterograde axonal transport to the LC axons that terminate at the CMN. Anterograde metal transport has been described for a variety of metal [41]; (6) the Hg^2+^-thiol complex is released at the LC axon’s CMN terminal, either at typical synapses, or at non-synaptic release sites in the vicinity of the CMN [42]; (7) the Hg^2+^-thiol complexes are transported into the CMN, possibly by molecular mimicry involving a vesicular transporter [43] or because cysteine is taken up avidly by neurons to form glutathione [44]. The other major form of toxic metal transport, ionic mimicry, is less likely to occur with Hg^2+^ since in biological systems Hg^2+^ is usually bound to thiol-containing molecules [42].

**Figure 4:**
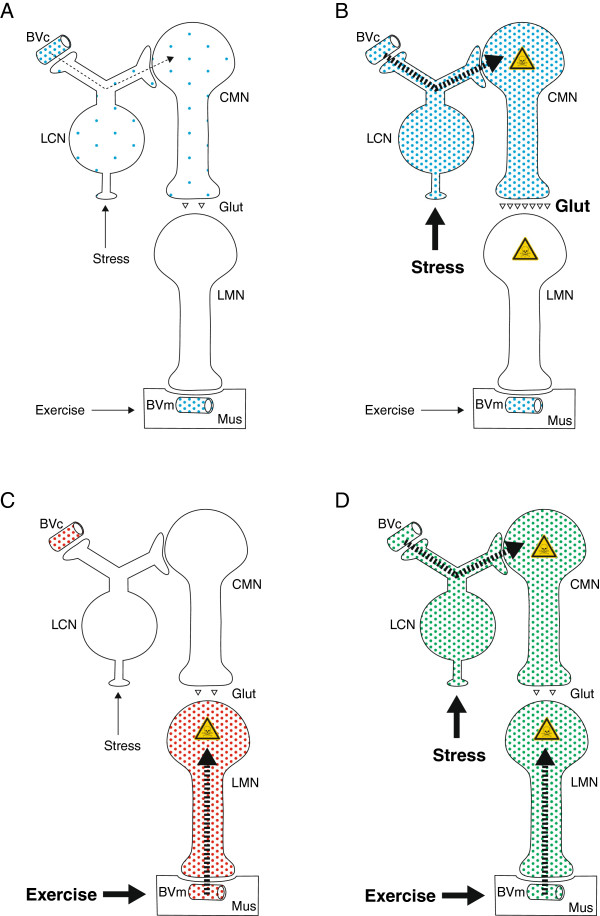
**Potential pathways for toxins to enter motor neurons and the possible resulting ALS phenotypes.** (**A**) This circulating toxin is taken up from cerebral (but not peripheral) blood vessels and enters LC neurons. The LC neurons then pass the toxin on to CMNs (thin dashed arrow). With low levels of stress, CMNs take up only a small amount of the toxin, which can readily be handled by cellular detoxifying mechanisms. (**B**) Under high levels of stress, increased noradrenaline recycling in LC neurons leads to a greater uptake of the toxin into CMNs, which overwhelms the cellular detoxifying mechanisms and could cause an upper motor neuron predominant form of ALS. A toxin that causes CMNs to produce more glutamate will damage LMNs, possibly leading to classical ALS. (**C**) During strenuous exercise an increased uptake of circulating toxin from intramuscular blood vessels at neuromuscular junctions damages LMNs. This could result in a lower motor neuron predominant form of ALS. (**D**) This toxin, aided by both stress and exercise, is taken up from both cerebral and intramuscular blood vessels and so can enter CMNs and LMNs. This could result in classical ALS. BVc: cerebral blood vessel, BVm: intramuscular blood vessel, CMN: corticomotor neuron, Glut: glutamate, Hg^AMG^: autometallographic-demonstrable mercury, LC: locus ceruleus, LFB: Luxol-fast blue, Mus: muscle.

Stressors (stimuli that disrupt homeostasis) can be divided into those that are physical, psychological, social, and those that disrupt cardiovascular or metabolic homeostasis [37]. NA output from the LC of animals increases markedly after exposure to a large number of different stressors, such as cold, restraint, electric shocks, chronic social stress, and forced walking [45]. If inorganic mercury enters CMNs via the re-uptake of NA into the LC, it is likely that this stressor-activated path-way deposits a large amount of mercury into CMNs (Figure 4B), more than can be detoxified by binding to metallothionein [38]. Our patient committed suicide, and although we do not have details of his state of mind in the weeks leading up to his death, it is to be expected that he would have been under considerable psychological stress. So of relevance is the finding that, compared to 11 controls, 6 suicide victims had fewer neurons in their LC when these were quantitated at *post mortem* examination [46]. This would be expected if stress-induced upregulation of NA output from the LC allowed circulating neurotoxins to enter and damage these neurons.

A stressor-induced increased toxin uptake into CMNs could explain the conundrum concerning the increased incidence of SALS in military personnel. Three studies of SALS in the US military, all using different methodologies, have reported a doubling of SALS incidence after military service [47-49]. One of these showed that military personnel who saw active (and presumably stressful) service had an increased risk of SALS compared to those who did not [48]. Of interest, all divisions of the military (army, navy and air force) had a increased risk of SALS, despite being engaged in different duties, suggesting some common factor underlay the increased risk of SALS. Although two of the studies involved Gulf war veterans [47,48], the third looked at servicemen before 1990 [49], which suggests that SALS risk is not related to any particular war zone, but to the experience of war itself. This led to criticism that such studies are unlikely to find any particular toxic agent responsible for the increased risk of SALS [50]. However, one thing these military personnel would have had in common is increased stress, with concomitant LC activation and the potential to take up any circulating neurotoxins that were present during the time of stress, which could then enter CMNs. Of note, decreased numbers of neurons in the LC have been reported in military personnel with post-traumatic stress disorder, a finding which would be expected if stress were promoting the uptake of toxins into this nucleus [51].

Epidemiological studies have identified a number of occupations which appear to be associated with an increased risk of SALS [8]. A large number of these occupations are stressful, such as truck drivers [52,53], airline pilots [54], and professional athletes and sportsmen such as soccer players [55]. One study has suggested an increased risk of SALS associated with self-reported stress [56], though another showed that the death of a child had no effect on the later risk of SALS [57]. It remains to be determined whether people who go on to have SALS have been subjected to increased stress at the same time that they have been exposed to environmental neurotoxins.

#### Via astrocytes

The possibility that astrocytes could take up metals from blood vessels and pass them on CMNs has been mentioned above.

#### Via the kidney

Recently it was shown that replicating viruses injected into mouse kidney can be transferred to CMNs via a chain of sympathetic neurons [21]. However, in our mercury-exposed mice no or very small amounts of mercury could be seen in sympathetic ganglia, and none was present in intermediolateral neurons, so mercury seems unlikely to use this route to reach CMNs.

#### Via lower motor neurons

CMNs make direct connections with LMNs in higher primates [27], and theoretically a toxic metal could pass between the two, as has been shown for tetanus toxin [58]. However, no significant mercury staining was seen in LMNs in our human case, so it seems unlikely that transfer of mercury from lower to upper motor neurons took place in this individual.

### Uptake and routes of metal transport into lower motor neurons

#### Animal

In our mice exposed to Hg^0^ or HgCl^2+^ mercury was found in LMNs but not in CMNs. Uptake of toxic metals into LMNs has been well studied in rodents [41]. The pathway appears to be from circulating blood into striated muscle, from where the metals are taken up at the neuromuscular junction, either via calcium channels in the case of lead [59] or by recycling vesicles [60]. From the axon terminal the metals are retrogradely transported to the LMN cell body [41].

#### Human

Our mercury-exposed human had no significant mercury in his LMNs, unlike the two previously reported human cases, both of whom had mercury in their spinal and brain stem motor neurons [12,13]. This could be because in our case the time between mercury exposure and death was only a few weeks, compared to the other two cases where this time was a number of years. These two cases would therefore have had considerably more time for mercury to be transported from their neuromuscular junctions to their LMNs. We do not know how physically active our individual was between his injection of mercury and death 5 months later. However, a period of relative inactivity might explain the lack of mercury in his LMNs. Exercise induces hypertrophy of the neuromuscular junction [61] and enhances neuromuscular transmission across the junction [62], both of which would facilitate toxin transfer into LMNs via the neuromuscular junction. A number of studies have reported that previous episodes of intense physical activity are associated with a greater susceptibility to ALS later in life [55,63-66]. This may be because exercise increases the chance of “suicide transport” [67] of circulating toxins into LMNs.

### Variations in the amount of toxins in motor neurons and different ALS phenotypes

Uptake of circulating toxins can vary between CMNs and LMNs for a number of reasons: (a) CNS blood vessels with tight junctions and peripheral vessels without tight junctions could take up different toxins (Figure 4A); (b) different recycling mechanisms at axon terminals (e.g., noradrenaline in LC neurons and acetylcholine in LMNs) could favor certain toxins; (c) some toxins could enter astrocytes adjacent to CNS blood vessels and their absence at the neuromuscular junction means that toxin could not enter via this route; (d) different physiological stimuli upregulate recycling mechanisms differently, e.g., stress upregulates LC neuron noradrenalin recycling and exercise upregulates acetylcholine recycling.

#### Toxins entering CMNs selectively: primary lateral sclerosis or ALS

A toxin taken up from CNS blood vessels by the LC would preferentially enter CMNs (Figure 4B). This would be enhanced by stressor upregulation of LC activity, which could pump more circulating toxin into CMNs. This surge of toxin could overcome the cell’s defence mechanisms and cause one of the many pathogenetic alterations described in heavy metal toxicity, in addition to protein misfolding. If only CMN damage occurred, the upper motor neuron form of ALS (primary lateral sclerosis) could result. However, if the toxin caused the CMN to produce more glutamate at its synapse with the LMN (possibly by astrocyte mercury exciting the CMN) the LMN could suffer excitotoxic death, resulting in a classical ALS phenotype. Compelling arguments have been put forward that ALS can be caused by cortical hyperexcitability [68] and recent neurophysiological evidence supports this concept [69].

#### Toxins entering LMNs selectively: progressive muscular atrophy

A circulating toxin that entered the LMN via the neuromuscular junction, possibly enhanced by increased exercise, could poison the LMN and result in the LMN-predominant form of ALS, progressive muscular atrophy (Figure 4C).

#### Toxins entering both CMNs and LMNs: ALS

A circulating toxin that was able to use both the CMN and LMN routes of entry (possibly aided by both stress and exercise) could poison both sets of neurons and result in an ALS phenotype (Figure 4D).

Although we have considered these phenotypic variations as they relate to SALS, in familial ALS marked variations also occur in the distribution of weakness and age of disease onset in different family members. In addition to gene-gene interactions, it is possible that having a variable toxin load in different sets of motor neurons makes these neurons differentially susceptible to ALS-associated genetic mutations.

## Conclusion

In conclusion, we have described the unique case of a man who would have been under psychological stress at the same time as he was exposed to inorganic mercury. At *post mortem* mercury was present in CMNs and LC neurons. We suggest that stress-related upregulation of LC neurons enabled large amounts of circulating mercury to be shunted into CMNs. While we have focused on heavy metal uptake, a range of environmental neurotoxins could just as readily use this “toxin-stress” pathway to enter CMNs. We have further shown differences between this human CMN uptake of toxin and the rodent uptake of circulating toxins into LMNs, which could be increased by exercise. Because ALS probably begins some years before symptoms become apparent, any search for environmental toxins that underlie the disease need to take into account life-long exposures. Finally, we suggest that phenotypic variations in both sporadic and familial ALS may be due to different amounts of toxins in upper and lower motor neurons.

## Methods

### Human exposed to metallic mercury

The detailed clinical information on this individual has been described previously [14]. Briefly, a 24 year-old man injected metallic mercury into his antecubital vein, from which he suffered no apparent ill effects, consistent with other cases of metallic mercury self-injection [70]. Five months later he died after committing suicide by lacerating both his wrists. At *post mortem* examination metallic mercury globules were present in his myocardium and lungs, but not in the brain. Tissue blocks were processed routinely for paraffin sections. For the present study, further 7 μm sections were cut from all available paraffin blocks (including those not stained in the previous study) of the cerebrum, cerebellum, brain stem, upper cervical cord and kidney. Paraffin sections from the same regions of a 26-year-old man who died from complications of cystic fibrosis were used as a control.

### Mice exposed to mercuric chloride or mercury vapor

6-week-old BALB/c mice (four per group) had been exposed previously to either: (a) a single dose of 2 μg/g intraperitoneal HgCl_2_, with sacrifice either 7 or 705 days later; (b) either 50 μg/m^3^ or 500 μg/m^3^ Hg^0^ for 4 h a day for 6 days, with sacrifice 7 days later. Control mice were given either an intraperitoneal saline injection or placed in the exposure chamber without Hg^0^ flow [36,71]. For the present study, further 7 μm sections of formalin-fixed paraffin-embedded blocks of brain, spinal cord and kidney were cut and stained for mercury, using the same protocol as for the human sections. Where necessary, further sections were cut to locate regions, for example the LC and sympathetic ganglia, that were not previously studied.

### Detection of mercury in tissues

Tissue was stained for mercury using AMG [15]. Briefly, paraffin sections were placed in physical developer containing 50% gum arabic, citrate buffer, hydroquinone and silver nitrate at 26°C for 80 min in the dark, then washed in 5% sodium thiosulphate to remove unbound silver. Sections were counterstained with either hematoxylin or Luxol-fast blue and viewed under bright-field illumination. Silver-coated mercury deposits are referred to as autometallography-demonstrable mercury (Hg^AMG^). In each staining run, a positive control section was included which contained mouse spinal motor neurons with known mercury deposits from a previous intraperitoneal injection of HgCl_2_.

Mercury staining within individual neurons was considered to be “heavy” if more than 10 Hg^AMG^ grains were seen, “light” if 3–10 grains were seen, and “absent” if fewer than 3 grains were seen. Mercury staining was considered to be “widespread” if more than 40% of neurons of the same type or within the same nucleus contained Hg^AMG^ grains, and “patchy” if 40% or fewer of these cells contained Hg^AMG^ grains. Mercury staining was considered to be present in glial cells if 2 or more Hg^AMG^ grains were seen in the cell.

The anatomical nomenclature used (for both human and mice neuroanatomy) was from the Allen Mouse Brain (http://mouse.brain-map.org.) and Spinal Cord (http://mousespinal.brain-map.org.) online atlases. The protocol for the human part of the study was approved by the Human Ethics Committee of the Sydney South West Area Health Service, and the animal protocol was approved by the University of Sydney Animal Ethics Committee.

## Abbreviations

BVc: Cerebral blood vessel; BVm: Intramuscular blood vessel; CMN: Corticomotor neuron; Glut: Glutamate; HgAMG: Autometallography-demonstrable mercury; LC: Locus ceruleus; LFB: Luxol-fast blue; Mus: Muscle; NA: Noradrenaline.

## Competing interests

The authors declare that they have no competing interests.

## Authors’ contributions

RP conceived the study, participated in its design, undertook its coordination and drafted the manuscript. SKJ participated in the study’s design and carried out the histochemistry. Both authors read and approved the final manuscript.
